# Litigation in access to universal health coverage for children and adolescents in Brazil

**DOI:** 10.3389/fpubh.2024.1402648

**Published:** 2024-06-25

**Authors:** Suely Coelho Tavares da Silva, Paulo Henrique Tavares da Silva, Robson Antão de Medeiros, Vânia Barbosa do Nascimento

**Affiliations:** ^1^Centro Universitário FMABC (FMABC), Santo André, Brazil; ^2^Centro Universitário de João Pessoa (UNIPÊ), João Pessoa, Brazil; ^3^Universidade Federal da Paraíba (UFPB), João Pessoa, Brazil

**Keywords:** universal health care, universal health coverage, child health, adolescent health, right to health

## Abstract

**Background:**

Brazil’s Unified Health System (SUS) ensures universal, equitable, and excellent quality health coverage for all. The broad right to health, supported by the Constitution, has led to excessive litigation in the public sector. This has negatively impacted the financial stability of SUS, created inequality in children and adolescents’ access to healthcare, and affected communication between the healthcare system and the judiciary. The enactment of Law Number 13.655 on 25 April 2018, proposed significant changes in judicial decisions. This study aimed to investigate decision-making changes in health litigation involving children and adolescents following the implementation of the new normative model.

**Methods:**

The study is cross-sectional, analyzing 3753 national judgment documents from all State Courts of Brazil, available on their respective websites from 2014 to 2020. It compares regional legal decisions before and after the promulgation of Law Number 13.655/2018. Data tabulation, statistical analysis, textual analysis, coding, and counting of significant units in the collected documents were performed. The results of data cross-referencing are presented in tables and diagrams.

**Results:**

The majority (96.86%) of legal claims (3635 cases) received partial or total provision of what was prescribed by the physician. The Judiciary predominantly handled these cases individually. The analysis indicates that the decisions made did not adhere to the norms established in 2018.

**Conclusion:**

Regional heterogeneity in health litigation was observed, and there was no significant variability in decisions during the studied period, even after the implementation of the new normative paradigm in 2018. Technical-scientific support was undervalued by the magistrates. Prioritizing litigants undermines equity in access to Universal Health Coverage for children and adolescents.

## Introduction

Children and adolescents face vulnerability regarding the violation of their fundamental rights, which is inherent to their developmental stage and whose health is disadvantaged by the low investments in public policies. In Brazil, the State has comprehensively protected this age group since the 1988 Constitution (1), particularly through Article 227, and further expanded these protections via the Statute of the Child and Adolescent in 1990. This statute represents a national achievement, ensuring priority and unrestricted access to healthcare policies within the Unified Health System (SUS) (2–4).

The United Nations Convention on the Rights of the Child, from 1989, in force in Brazil since 1990, formulated global norms for the protection of children’s rights, which apply to the development of laws, policies and all programs that address the guarantee of equity in the domains of child health practices. Above all, it emphasizes the maximum defense of those with greater vulnerability. The broad scope of the convention is to commit the States Parties to ensure that children receive priority assistance and access “to the highest possible standard of health” (5, 6).

Excessive personal healthcare expenses, exceeding 10% of household budgets, plunge millions of children and their parents into extreme poverty. According to the World Health Organization, “adequate and sustained health financing is a prerequisite for the achievement of Universal Health Coverage (UHC).” Despite this, service coverage declined, impacted by reductions in government funding and rising public health costs. Financial constraints on individuals and disruptions in routine healthcare services due to inability to pay now threaten the achievement of Sustainable Development Goals (SDGs) for health by 2030 (7). Middle and low income countries account for more than 90% of pediatric deaths, with more than 16000 deaths of children under five, per day. The number of preventable deaths among children is a parameter that reveals inequalities in health coverage (8, 9).

The concept of UHC in various countries entails utilizing public funds as the primary financing mechanism. The strategies employed to achieve UHC can encompass a spectrum from purely state-run services to collaborations involving both public and private entities, as well as direct contracts with private healthcare providers or social organizations (10). Universal coverage ensures quality health care and related services, promoting comprehensive well-being for individuals and communities. This underscores concerns about equity and the full implementation of the right to health, encompassing a multidimensional framework of humanitarian, economic, and financial protection (11, 12).

The Unified Health System provides free and universal access to medical care for children and adolescents in Brazil. However, political, and economic crises have undermined its scope and capacity to meet public demands, especially at the federal level, between 2014 and 2018, this resulted in a 3% reduction in health expenditure per person, in the consolidated of the three spheres of government (11, 13). Thus, for health care to be fully achieved, it is indispensable that the health system functions perfectly, with the guarantee of the provision of essential resources. In the impossibility of meeting these minimum requirements, that the mechanisms appropriate to the repair of the rights neglected by the State be imposed by law, provided that fair and ethical spending, the responsibility of the health system are safeguarded (14–16).

Healthcare judicialization—legal claims to guarantee public access to health technologies—has further strained SUS sustainability, resulting in access imbalances (10, 17, 18). Legal actions demand already incorporated medications, procedures, and other prescribed technologies, according to the access criteria regulated by national Clinical Protocols and Therapeutic Guidelines, which were either unavailable in health services or not funded by SUS, as well as those not included in the lists provided by Federal, State, and Municipal Health entities (17). Thus, economic conflicts arise, typical of litigation in health, since the compromise of the budget allocation, destined to ensure the demands of the population as a whole, vs. the fulfillment of the particular request of the litigant, usually associated with high-cost treatments, items not authorized by the National Health Surveillance Agency or that are not included in the lists of procedures or medications provided by SUS (19, 20).

Health litigation by providing a platform for citizens to voice their grievances, signals deficiencies in the healthcare system. The healthcare system rarely gathers information from the judiciary. The limited interaction between both systems creates communication gaps, except in cases requiring coercive directives to provide specific services (21). Thus, the process of judicialization of health results in a determination that should be obeyed by public managers, and marks an interrelation between the Judiciary and the Executive, which is not always fulfilled. It should be emphasized that the Judiciary should have an exceptional role in guaranteeing the right to health to those who claim it, and that it is up to the states to organize and administratively provide the right to health, in the field of public policies (22).

It should be noted that magistrates, in the period prior to the validity of Law No. 13.655/2018, immediately ordered the supply of the item involved in the dispute, based on the support of the right to health, guaranteed by the Constitution, and qualified as “insufficient reasons” to deny them, the limited budgetary condition of the State, the lack of provision of expenses, or even the absence of the drug demanded in the lists of SUS availability (23).

The Law Number 13.655/2018 brought significant changes to the Law of Introduction to Brazilian Norms. This legislation now requires judges, in decisions about public health, to incorporate economic difficulties, management obstacles, and health policy requirements to promote a collaborative relationship between public administration and the judiciary, providing perspectives to mitigate the risks of financial collapse for both litigants and the health system (24), as well as the sanctioning of healthcare system managers when they fail to comply with a court ruling (25).

The effectiveness of this new normative paradigm (Law 13.655) raises questions about its impact on decision-making in health litigation in Brazil, especially regarding access to UHC for children and adolescents. The hypothesis is that despite being viewed as progressive, legislative changes have not fully integrated into the judiciary’s practice. The judiciary maintains a model focused on individual demands based on isolated legal principles and lacks meaningful dialog with public entities.

This study aims to assess whether the judiciary altered its decision-making approach in cases related to the healthcare rights of children and adolescents, responding to the normative shift initiated by Law Number 13.655/2018. The research has specific objectives:

- Identify characteristics of healthcare-related judicial claims of children and adolescents in Brazil during the studied period;
- Calculate the proportion of individual and public civil actions adjudicated, distributed across research regions in Brazil;
- Assess the presence or absence of technical opinions in judges’ decision-making based on scientific evidence;
- Compare magistrates’ decisions, focusing on indicators of universality, quality, and equity, before and after Law Number 13.655/2018; and
- Determine if Brazilian magistrates have adopted the new normative paradigm in judicial decisions.

## Methods

This cross-sectional study utilizes descriptive analysis, employing both quantitative and qualitative methods. It focuses on publicly available legal decisions from both state courts and the Federal District court in Brazil, specifically concerning healthcare technology involving children and adolescents through the SUS. The study covers cases adjudicated between 01 March 2014 and 28 February 2020. For the quantitative analysis, all publicly available case summaries and/or their corresponding full decisions from the specified period were included. The search criteria used keywords like “children and adolescents” and “provision of medications or health treatment,” within the context of individual-judge decisions and public civil actions. Duplicate summaries, those lacking comprehensive information, or related to decisions not fully available or processed confidentially were excluded.

Indicators were established to monitor the implementation of universal healthcare coverage, based on the premise that judicial decisions should guarantee equitable and universal access to essential, effective, and high-quality healthcare technologies for all citizens while safeguarding the integrity of the healthcare system. Consequently, the indicators include universality, defined as the rate of claims processed and approved by the judiciary; quality of judicial decisions, defined as the percentage of decisions based on scientific evidence and, equity in decision-making, defined as the proportion of decisions incorporating the normative paradigm, established by Law Number 13.655/2018.

There were 3753 judicial decisions extracted from the database (26). Nominal variables were categorized for statistical calculations using the free IBM SPSS (RRID:SCR_002865) for Windows, subscription ID 508677891. Descriptive statistics were gathered for aspects like origin, required technology, types of legal actions, outcomes of judicial decisions, and technical-scientific support. Data, grouped by Brazilian regions, was divided into two samples based on the decision date, spanning from 01 March 2014 to 25 April 2018, and from 26 April 2018 period prior to the validity of Law 13.655, and from 26 April 2018 to 28 February 2020, period after the enactment of said Law.

First, the null hypothesis of no statistical difference between the groups and the alternative hypothesis of having a statistical difference were established. Then, a statistical test was selected to compute a standardized numerical measure of the difference between the groups. The techniques of regression analysis are the most widely used statistical tools for discovering the relationships among variables using real-world data and assessing their validity (27, 28).

A multiple regression analysis was conducted with the decision date as the dependent variable. Due to the relatively low explanatory power of R^2^, two regression models were chosen based on the specified periods to validate the distribution of the mean of the two samples. In these models, the dependent variable was the territorial unit, divided into Brazilian regions—North, Northeast, Midwest, South and Southeast. Model 1, corresponding to the period until 25 April 2018, and model 2, to the period from 26 April 2018, were considered. For both, the significance levels: 0.001; 0.01; 0.05; 0.1, which indicate the probability of observing a difference as large or larger than the one observed under the null hypothesis of no statistical difference between the groups.

In these models, the dependent variable was the territorial unit, divided into regions. The significance levels used were 0.001, 0.01, 0.05, and 0.1. The coefficients of determination for the regression models were established, with the Multiple Correlation Coefficient serving as a measure of the predictive quality of the dependent variable, which is the territorial unit divided by regions, for both models. A confirmatory analysis of variance (ANOVA) test was applied, and since the *p*-value was nearly zero, the null hypothesis of equal means was dismissed at all significance levels.

The qualitative analysis compared judicial decisions before and after the implementation of Law Number 13.655/2018 to validate the hypothesis, generating a more complete perspective of the phenomena analyzed in the quantitative phase of the research. The SPICE-model for formulating a research question for qualitative studies (S stands for setting, P for perspective, I for intervention, C for comparison, and E for evaluation) was more appropriate for a research question explored in the analysis—The effectiveness of this new normative paradigm (Law 13.655) impact on decision-making in health litigation in Brazil, especially regarding access to universal health coverage (UHC) for children and adolescentes? (29).

The study included 74 documents, selecting judicial decisions guided by information saturation needs, when the repetition of the analyzed information is perceived, considering the most significant ones from each court, for the understanding of the analyzed phenomenon, propelling insights, which guarantee significance in the debate of relevant aspects of the qualitative study (30). “In qualitative research, there are no rules about what size a sample needs to be; instead this is generally determined by the need for information” (31).

The content analysis of the judicial decisions was developed following three stages predicted by Laurence Bardin (32):

- pre-analysis—reading and selecting the most relevant collected documents;
- coding of the registration units, represented by words or legal expressions, distributed according to the context in which they relate, followed by the categorization of the material in two moments, before and after the implementation of Law 13.655/2018; and
- treatment of the results obtained and interpretation.

The coding and counting of meaningful units were conducted using Nvivo (RRID:SCR_014802) 12 Pro Student software. The registration units represented by words with five or more letters composed a word cloud, whose visual representation indicates the frequency and importance of these in the context of the analysis carried out.

Content analysis identified principal arguments within the decisions, interpreted using a theoretical framework. The arguments were transcribed, and key phrases were extracted, enabling the visualization of similar semantic patterns. Record units were created based on similarities and differences among these phrases, facilitating the identification of central ideas, subsequently categorized to assess the direction and intensity of the co-occurrence of meaningful units (Swedish Agency for Health Technology Assessment and Assessment of Social Services, 2016). The measure of intensity evaluates values/trends (32), while the direction assesses whether attitudes are positive, negative, neutral, or mixed.

**Figure 1:**
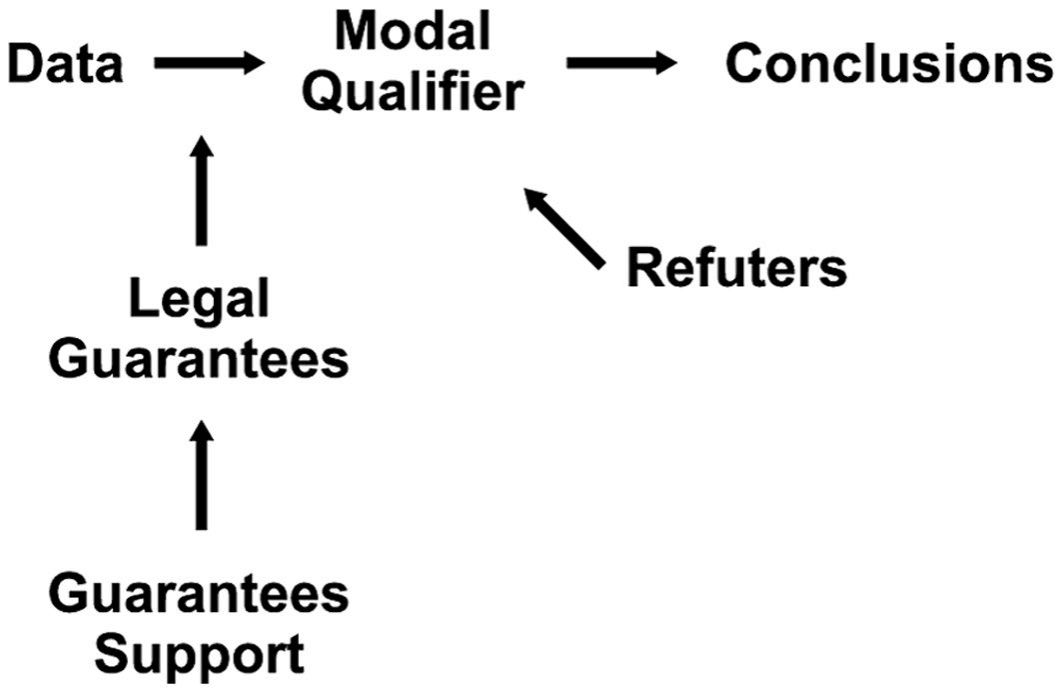
Toulmin model of arguments (33).

The study adapted the techniques of legal reasoning analysis on practical argumentation for its purposes. To organize and analyze the argumentation extracted from legal decisions, the argumentative reasoning model postulated by Toulmin was chosen (33, 34). This model allows for the validation of the verdict presented by the magistrate, which may indicate rejection or total or partial guarantee of the disputed object. The arrows indicate the relationship between the elements in the layout. The data alleged by the claimant serve as support for the conclusion. The Modal Qualifier, positioned next to the conclusion, indicates the claims that reinforce it. The refuters, just below, establish conditions capable of invalidating the claims. Legal Guarantees confer legitimacy to the litigant’s arguments, and their credibility is supported by infraconstitutional laws (Figure 1).

## Results

### Quantitative analysis

The data collection yielded 4120 sentences. After a comprehensive review, cases (*n* = 365) not specifying judicial claims related to healthcare and cases not related to the SUS (*n* = 2) were excluded. None of the sentences were subjected to post-ruling confidentiality orders. This resulted in a final sample size of 3753 sentences from state and federal district courts, distributed across Brazilian regions based on the nature of healthcare judicial claims (26). Notably, there was a higher frequency of disputes in urban areas in the South region, while a lower frequency was observed in State Law Courts with lower population density in the Central-West and North.

The distribution pattern, in numerically descending order of demands, was heterogeneous across the regions of the Federation. However, requests for therapeutic purposes, such as medications or other pharmaceutical products and appointments with physicians or specialists in various fields and surgical procedures prevailed in the overall aggregate. There was no predominant focus on specific drugs or therapies to treat rare diseases. Nutritional purposes related to infant feeding, special infant formulas, and consultations with nutritionists followed in numerical sequence. Requests that encompassed more than one demand object accounted for 5.17% of the total and occupied the third position (Table 1).

**Table 1:** Purposes of the healthcare judicial claims involving children and adolescents to the SUS (*n* = 3753), distributed across Brazilian regions.

Purposes of healthcare judicial claims (*n* = 3753)	Region											
	North	%	North-east	%	South	%	Central-West	%	South-east	%	Total	%
Therapeutic	337	8.98	486	12.95	736	19.61	250	6.66	502	13.38	2311	61.58
Nutritional	103	2.74	221	5.89	189	5.04	81	2.16	116	3.09	710	18.92
More than one	26	0.69	54	1.44	40	1.07	21	0.56	53	1.41	194	5.17
Diagnosis	44	1.17	47	1.25	28	0.75	21	0.56	15	0.40	155	4.13
Rehabilitation	11	0.29	44	1.17	51	1.36	7	0.19	33	0.88	146	3.89
Supplies	9	0.24	45	1.20	26	0.69	11	0.29	19	0.51	110	2.93
Prevention	2	0.05	17	0.45	15	0.40	41	1.09	7	0.19	82	2.18
Hygiene	9	0.24	17	0.45	6	0.16	4	0.11	3	0.08	39	1.04
Transport	2	0.05	0	0.00	0	0.00	0	0.00	0	0.00	2	0.05
Palliative	0	0.00	0	0.00	0	0.00	1	0.03	0	0.00	1	0.03
General Care	0	0.00	0	0.00	1	0.03	0	0.00	0	0.00	1	0.03
Not informed	1	0.03	1	0.03	0	0.00	0	0.00	0	0.00	2	0.05
Total	544	14.50	932	24.83	1092	29.10	437	11.64	748	19.93	3753	100

In terms of pediatric medical specialty demand in Brazil, neurology and neurosurgery were predominant (22.54%; *n* = 846), particularly in the South. This was closely followed by nutrition (19.00%; *n* = 713), which was more prevalent in the Northeast. Endocrinology was also in high demand (12.92%; *n* = 485), except in the North region. Psychiatry was highly sought after (9.25%; *n* = 347) in the Northeast, Southeast, and South regions. Socioeconomic disparities were relevant aspects in regional availability for medical specialties, with marked differences between the more impoverished North and the more prosperous South, as well as divergences within urban and rural areas (Table 2).

**Table 2:** Judicial claims for pediatric medical specialties involving children and adolescents to the SUS (*n* = 3753), distributed across Brazilian regions.

Medical specialty	Regions											
	North	%	North–east	%	South	%	Central-West	%	South-east	%	Total	%
Neurologist/Neurosurgeon	109	2.90	206	5.49	283	7.54	86	2.29	162	4.32	846	22.54
Nutritionist	103	2.74	221	5.89	191	5.09	80	2.13	118	3.14	713	19.00
Endocrinologist	0	0.00	124	3.30	108	2.88	52	1.39	133	3.54	485	12.92
Psychiatrist	29	0.77	54	1.44	116	3.09	25	0.67	123	3.28	347	9.25
Multidisciplinary Team	17	0.45	49	1.31	29	0.77	19	0.51	39	1.04	153	4.08
Allergist/Immunologist	12	0.32	19	0.51	24	0.64	49	1.31	18	0.48	122	3.25
Hepatologist	18	0.48	12	0.32	41	1.09	8	0.21	12	0.32	91	2.42
Otorhinolaryngologist	15	0.40	24	0.64	16	0.43	16	0.43	15	0.40	86	2.29
Pulmonologist	12	0.32	13	0.35	42	1.12	5	0.13	13	0.35	85	2.26
Surgeon	14	0.37	23	0.61	19	0.51	15	0.40	10	0.27	81	2.16
Orthopedist	17	0.45	24	0.64	20	0.53	11	0.29	6	0.16	78	2.08
Physiotherapist	11	0.29	9	0.24	41	1.09	2	0.05	11	0.29	74	1.97
Ophthalmologist	13	0.35	15	0.40	21	0.56	12	0.32	10	0.27	71	1.89
Pediatrician	10	0.27	24	0.64	21	0.56	2	0.05	11	0.29	68	1.81
Dermatologist	4	0.11	10	0.27	26	0.69	7	0.19	14	0.37	61	1.63
Cardiologist	20	0.53	14	0.37	8	0.21	5	0.13	5	0.13	52	1.39
Nephrologist	13	0.35	8	0.21	11	0.29	7	0.19	6	0.16	45	1.20
Geneticist	5	0.13	22	0.59	2	0.05	5	0.13	4	0.11	38	1.01
Hematologist	9	0.24	6	0.16	12	0.32	7	0.19	2	0.05	36	0.96
Oncologist	8	0.21	9	0.24	8	0.21	2	0.05	6	0.16	33	0.88
Neonatologist	2	0.05	13	0.35	11	0.29	2	0.05	3	0.08	31	0.83
Urologist	9	0.24	2	0.05	3	0.08	7	0.19	8	0.21	29	0.77
Rheumatologist	8	0.21	7	0.19	6	0.16	3	0.08	3	0.08	27	0.72
Speech Therapist	2	0.05	8	0.21	10	0.27	0	0.00	5	0.13	25	0.67
Intensivist	3	0.08	8	0.21	7	0.19	5	0.13	2	0.05	25	0.67
Vascular Surgeon	2	0.05	2	0.05	8	0.21	3	0.08	4	0.11	19	0.51
Infectiologist	4	0.11	4	0.11	6	0.16	0	0.00	1	0.03	15	0.40
Gynecologist	1	0.03	0	0.00	0	0.00	1	0.03	1	0.03	3	0.08
Dentist	0	0.00	0	0.00	1	0.03	1	0.03	0	0.00	2	0.05
Psychologist	0	0.00	0	0.00	0	0.00	0	0.00	1	0.03	1	0.03
Occupational Therapist	0	0.00	0	0.00	0	0.00	0	0.00	1	0.03	1	0.03
Nurse	0	0.00	0	0.00	1	0.03	0	0.00	0	0.00	1	0.03
Not informed	7	0.19	3	0.08	0	0.00	0	0.00	1	0.03	7	0.19
Total	544	14.50	932	24.83	1092	29.10	437	11.64	748	19.93	3753	100

To validate the variability in the mean distribution of the two samples, categorized by judgment date, two multiple regression analyses were conducted. In both models, the explanatory/dependent variable was stratified by Brazil’s regions, while the independent variables varied in each model. In Tables 3, 4, the multiple correlation coefficient indicated a high level of prediction for both models. The determination coefficient of Model 1 (R^2^ = 0.927) suggests that the variables were significant; the independent variables explained 92.7% of the variation in the dependent variable. In Model 2, we achieved an R^2^ of 0.931, indicating that the model’s explanatory power is 93.1%. The ANOVA test confirmed regional heterogeneity, highlighting the impact of diverse characteristics of judicialization based on origin.

**Table 3:** Regression coefficients for Model 1 (cases adjudicated up to 25 April 2018).

Variables	Estimate	Standard deviation	t-value	Pr(>|t|)	Level of significance
Intercept	4.66E+02	1.26E+02	3.682	0.000237	0.001
State of Origin	1.75E+02	1.11E+00	156.684	<2e−16	0.001
City of Origin	4.37E-03	3.21E-02	0.136	0.891714	
Age Group	-3.16E-01	3.58E-01	−0.882	0.377695	
Speciality of the Claim	1.69E-01	1.11E+00	0.153	0.878307	
Purpose of the Claim	2.69E-01	3.57E-01	0.753	0.451402	
Type of Action	3.30E-01	3.58E-01	0.922	0.356529	
Nature of the Case	-5.83E+00	1.35E+00	−4.329	1.57e−05	0.001
Technical-Scientific Opinion	-7.73E+01	3.24E+01	−2.387	0.017062	0.05
Court Decision Outcome	9.35E+01	1.89E+01	4.957	7.71e−07	0.001
Technical-Scientific Support	-1.72E+02	4.95E+01	−3.466	0.000539	0.001
Type of Technology	4.29E-01	3.42E-01	1.257	0.208834	

**Table 4:** Multiple regression coefficients for Model 2 (cases adjudicated from 26 April 2018 onwards).

Variables	Estimate	Standard deviation	t-value	Pr(>|t|)	Level of significance
Intercept	-3.96E+01	1.55E+02	−0.255	0.7985	
State of Origin	1.70E+02	1.33E+00	127.838	<2e−16	0.001
City of Origin	-1.11E-01	3.75E-02	−2.943	0.0033	0.01
Age Group	1.04E+02	2.24E+01	4.638	3.81e-06	0.001
Speciality of the Claim	-3.01E+00	1.30E+00	−2.317	0.0206	0.05
Purpose of the Claim	8.84E+00	4.37E+00	2.024	0.0432	0.05
Type of Action	5.58E+01	2.32E+01	2.405	0.0163	0.05
Nature of the Case	-5.66E-02	1.40E+00	−0.040	0.9678	
Technical-Scientific Opinion	9.39E+01	4.18E+01	2.246	0.0249	0.05
Court Decision Outcome	3.44E+01	2.07E+01	1.661	0.0968	0.1
Technical-Scientific Support	-3.18E+01	5.38E+01	−0.591	0.5544	
Type of Technology	-1.60E-01	3.43E-01	−0.465	0.6420	

Table 5 summarizes the case law profile, with 93.69% of applicants (3516 cases) granted full access. A total of 96.86% had successful claims, including 119 (3.17%) who partially obtained their claims. Judges often rule in favor of the plaintiff and grant the filed requests. Partial provision implies the need for personal expenditure to secure the item that could not be obtained in the dispute. A total of 2209 cases (58.86%) were adjudicated in state courts up to 25 April 2018, and 1544 cases (41.14%) were adjudicated from 26 April 2018 onwards. There is a marked disparity between the courts in the Central-West and North regions, adjudicating fewer cases and together accounting for just over 25% of the decisions, compared to the South, Northeast, and Southeast regions, which together adjudicated over 70% of the total.

**Table 5:** Case law concerning the health rights of litigants aged 0 to 18 (*n* = 3753), between 2014 and 2020, categorized by judgment date and Brazilian regions.

Case Law (*n* = 3753)	Regions											
	North	%	North–east	%	South	%	Central-West	%	South-east	%	Total	%
**Actions adjudicated up to 25 April 2018**												
Private Action	294	7.83	557	14.84	489	13.03	206	5.49	320	8.53	1866	49.72
Public Civil Action	51	1.36	87	2.32	102	2.72	50	1.33	46	1.23	336	8.95
Not informed	0	0.00	1	0.03	4	0.11	1	0.03	1	0.00	7	0.19
**Technical-scientific opinions**												
Non-existent	334	8.90	638	17.00	541	14.42	136	3.62	339	9.03	1988	52.97
Existent	11	0.29	7	0.19	54	1.44	121	3.22	27	0.72	220	5.86
Not informed	0	0.00	1	0.03	0	0.00	0	0.00	0	0.00	1	0.03
**Outcome of judicial decisions**												
Fully granted	332	8.85	622	16.57	583	15.53	244	6.50	304	8.10	2085	55.56
Rejected	7	0.19	7	0.19	1	0.03	10	0.27	26	0.69	51	1.36
Partially granted	6	0.16	15	0.40	7	0.19	3	0.08	31	0.83	62	1.66
Dismissed	0	0.00	1	0.03	4	0.11	0	0.00	5	0.13	10	0.27
Not informed	0	0.00	1	0.03	0	0.00	0	0.00	0	0.00	1	0.03
**Judicial decision backed by technical-scientific evidence**												
Yes	10	0.27	4	0.11	7	0.19	50	1.33	15	0.40	86	2.29
No	335	8.93	641	17.08	588	15.67	207	5.52	351	9.35	2122	56.54
Not informed	0	0.00	1	0.03	0	0.00	0	0.00	0	0.00	1	0.03
**Actions adjudicated from 26 April 2018**												
Private Action	135	3.60	222	5.92	409	10.90	154	4.10	332	8.85	1252	33.36
Public Civil Action	63	1.68	64	1.71	88	2.34	26	0.69	50	1.33	291	7.75
Not informed	1	0.03	0	0.00	0	0.00	0	0.00	0	0.00	1	0.03
**Technical-scientific opinions**												
Non-existent	189	05.04	282	7.51	462	12.31	105	2.80	329	8.77	1367	36.42
Existent	10	0.27	4	0.11	35	0.93	75	2.00	53	1.41	177	4.72
Not informed	0	0.00	0	0.00	0	0.00	0	0.00	0	0.00	0	0.00
**Outcome of judicial decisions**												
Fully granted	187	4.98	281	7.49	469	12.50	156	4.16	338	9.01	1431	38.13
Rejected	4	0.11	3	0.08	12	0.32	13	0.35	18	0.48	50	1.33
Partially granted	5	0.13	2	0.05	14	0.37	10	0.27	26	0.69	57	1.52
Dismissed	3	0.08	0	0.00	2	0.05	1	0.03	0	0.00	6	0.16
Not informed	0	0.00	0	0.00	0	0.00	0	0.00	0	0.00	0	0.00
**Judicial decision backed by technical-scientific evidence**												
Yes	6	0.16	3	0.08	22	0.59	34	0.91	34	0.91	99	2.64
No	193	5.14	283	7.54	475	12.66	146	3.89	348	9.27	1445	38.50
Not informed	0	0.00	0	0.00	0	0.00	0	0.00	0	0.00	0	0.00
Total	544	14.50	931	24.81	1092	29.10	437	11.64	748	19.93	3753	100

In judgments predating Law No. 13.655/2018, the Northeast had more ordinary actions, while the Southern region had a higher proportion of decisions against public administration in both ordinary and public civil actions. In the South, post-law enactment, there were more judgments for both ordinary and public civil actions. The Southeast ranked second for ordinary actions, and the Northeast for public civil actions. The judiciary primarily used individual proceedings in ordinary actions, unlike public civil actions, which have minimal impact on public policies.

The technical-scientific opinion (TSO) was sparingly used nationwide during the study, cited in only 10.5% of decisions. The Central-West region used technical-scientific information more frequently, while the Northeast made the least reference to its use. The Southeast secured the second position in supporting magistrates backed by TSO from 26 April 2018. However, the total of judicial decisions backed by technical-scientific evidence was negligible in all states during the specified periods, reaching 2.29% and 2.64%, respectively, contrary to expectations. Despite magistrates in the Central-West and Southeast more frequently making decisions based on TSO, these were the two regions that most often chose to reject plaintiffs’ requests.

### Qualitative analysis

**Figure 2:**
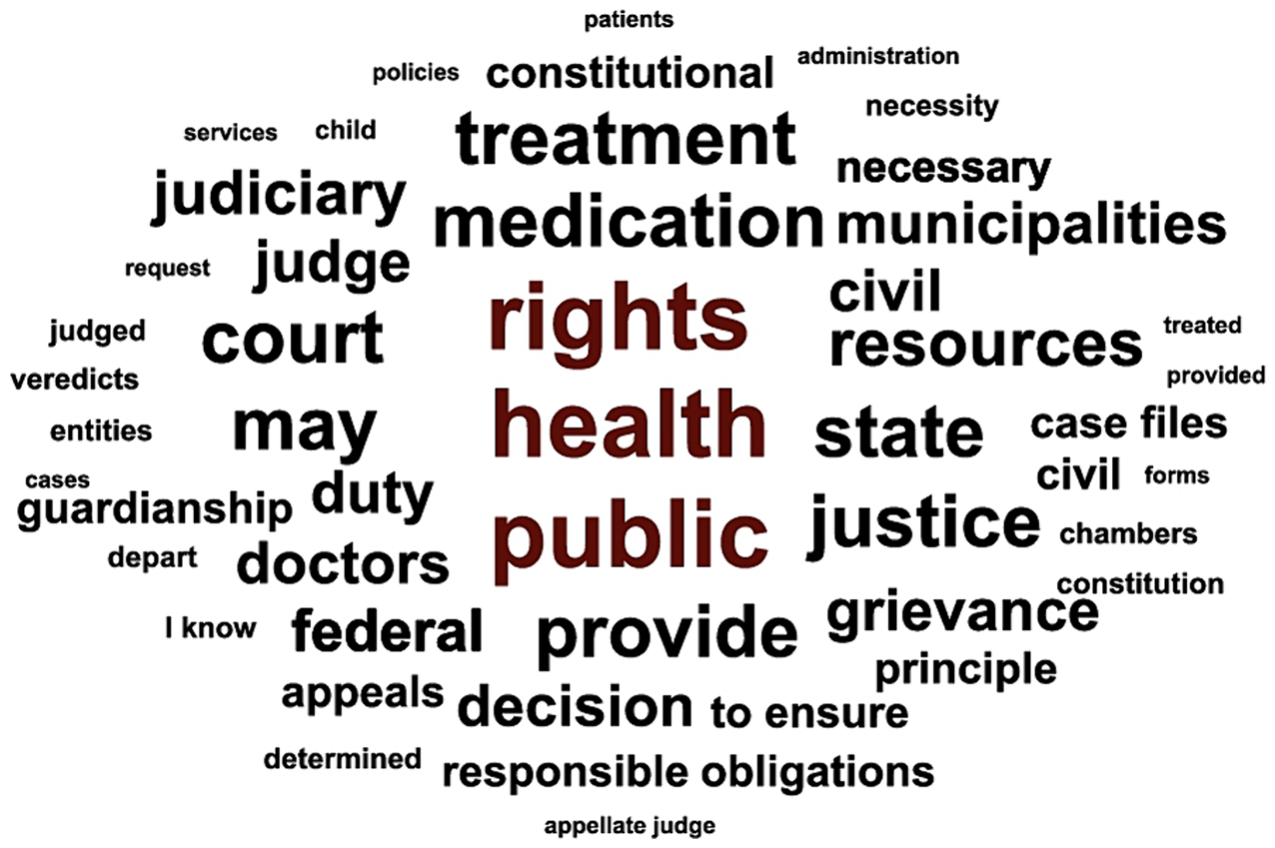
Illustrative word cloud of the top 50 frequently used terms based on document analysis.

A word cloud with correlated terms was generated through N-Vivo qualitative analysis (Figure 2). In the texts of the selected judicial decisions, a search for words with five or more letters resulted in the 50 most frequently mentioned terms. Among the top 10 terms, the word “resources” appeared prominently, strongly associated with the economic vulnerability of those involved in the judicial decisions.

The analysis of legal documents in search of essential elements did not find mentions of the terms “Law 13.655,” or related terms such as “legal implications of the decision,” “administrative implications,” and “Introduction to Brazilian Legal Norms Act.” The textual analysis revealed arguments such as the “principle of the reserve of the possible,” the “existential minimum,” and “financial resources,” with 51, 56, and 63 mentions, respectively. These legal expressions are relevant for decision-making and refer to the protection of economic priorities in the face of financial vulnerabilities for both parties involved in health-related legal disputes.

The arguments that structured the matrix of total acceptance of requests, the partial acceptance of requests, and the rejection of requests were analyzed in two sections, comparing the content of decisions judged up to 25 April 2018, with decisions judged from 26 April 2018, according to the Toulmin model (33), organized with the following elements: Data (alleged by the claimants); Conclusion (Total accepted, Partial accepted, Rejection of the requests); Modal Qualifier (favorable theses); Refutations (unfavorable theses); Warrant (constitutional guarantees); and Backing (infraconstitutional reinforcement). Thus, the trends identified in the intersection of information revealed that there was no significant variability in the judicial argumentation between the periods before and after the enactment of Law 13.655/2018.

**Figure 3:**
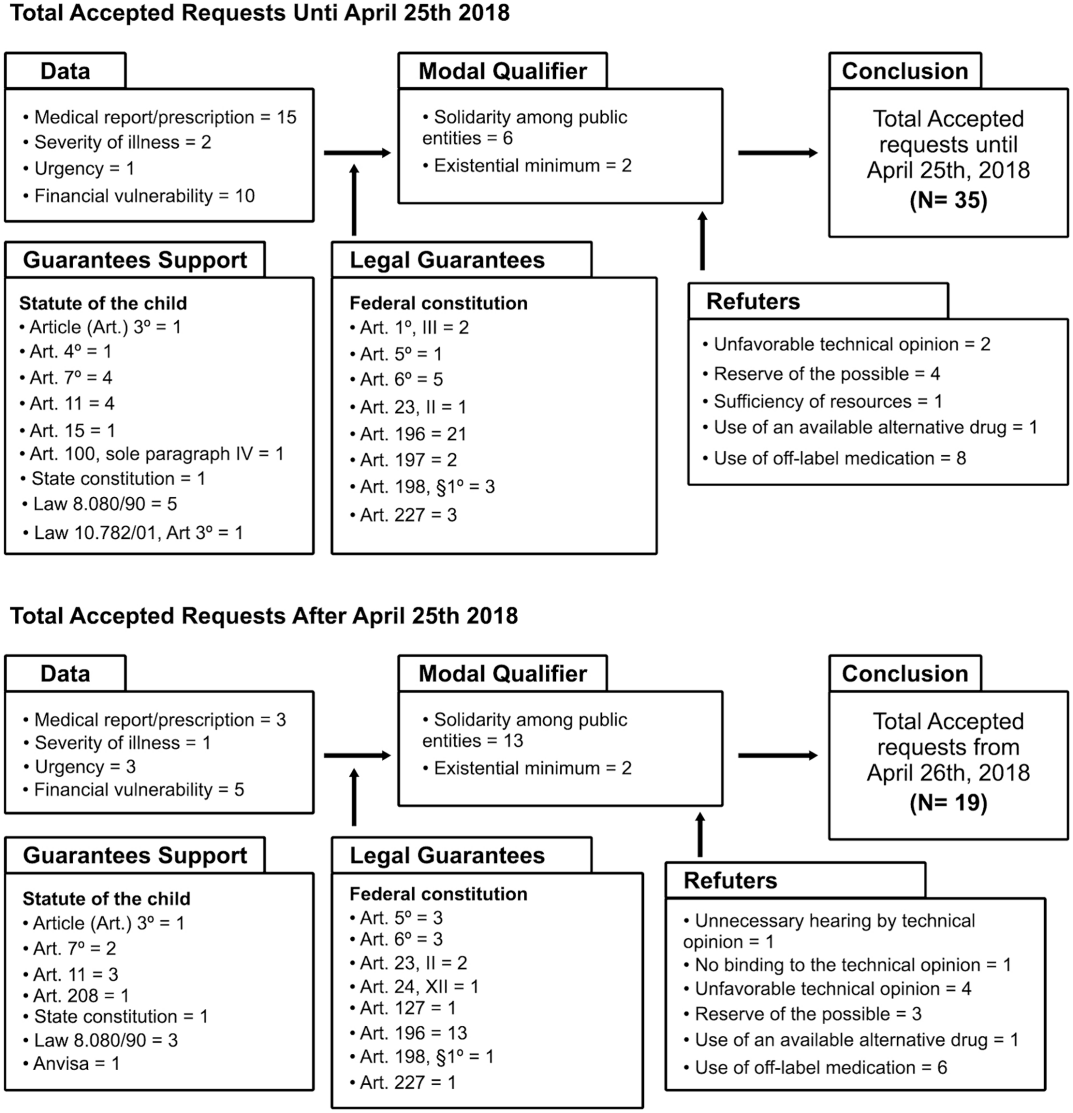
Argumentative matrix of total accepted requests based on judgment date.

The Argumentative Matrix of Total Accepted Requests (Figure 3) comprised *N* = 35 decisions judged until 25 April 2018, and *N* = 19 decisions judged from 26 April 2018. Most of the claimants asserted financial vulnerability, presenting medical prescriptions, reinforced by the qualifiers of solidarity among entities and existential minimum. Favorable decisions were supported by fundamental constitutional principles: dignity of the person (Art.1); inviolability of the right to life (Art. 5); social rights (Art. 6); protection of persons with disabilities (Art. 23); right to health (Art. 196 and 197); SUS guidelines (Art. 198); the duties of the Public Prosecutor (Art. 127); the duties of the family, society, and the State to ensure priority for children, adolescents, and youth (Art. 227); and infraconstitutional devices: Statute of the Child and Adolescent, State Constitution, Autism Spectrum Disorder Law, and SUS Law 8080/90. The Law 13.655/2018 was not mentioned. The technical opinion appeared as an unfavorable and unnecessary element. The frequencies of those expressions relevant to the construction of arguments that appeared in the analyzed judicial texts were recorded.

In the argumentative matrix of partially acceptance of demands *N* = 5 judicial decisions were analyzed in both temporal situations, before and after the enactment of the Law 13.655/2018. The arguments used to support the partial acceptance of demands were like those used for full acceptance.

**Figure 4:**
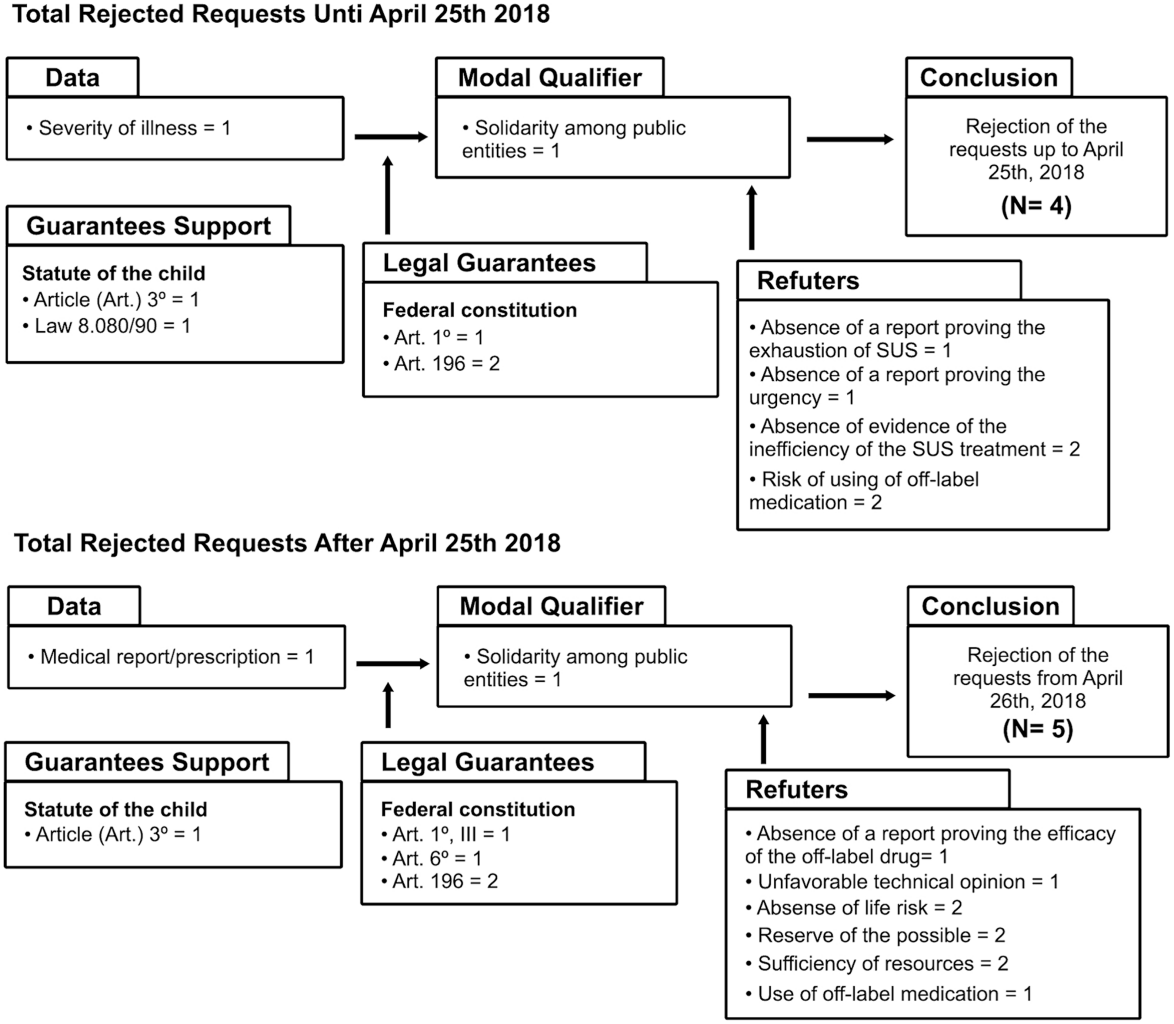
Argumentative matrix of rejected requests based on judgment date.

In the rejection matrix (Figure 4), were analyzed *N* = 4 judicial decisions before the enactment of the Law, until 25 April 2018, and from 26 April 2018, *N* = 5 decisions were judged. The following elements exhibited refutative behavior: lack of proof of inefficiency in SUS treatment, risk of off-label drug use, absence of life risk, reserve of the possible, sufficiency of resources, and unfavorable technical opinion. This implies that they were relevant for the refusal of demands, although this occurred in a small number of litigations. Some constitutional principles and infraconstitutional supports were used sparingly, but the Law 13.655 was not mentioned. Arguments associated with financial risk and solidarity among federated entities were decisive in denying the right to health, similarly in the two defined periods.

## Discussion

Regional litigation frequency aligns with existing literature, indicating a concentration of disputes in the Southeast and South regions. This is likely due to medicine shortages, uneven availability of specialized medical professionals, and demographic factors. Around 60% of Brazil’s impoverished population resides in urban centers, where almost half of the children come from low-income families. In 2015, poverty rates were 3.4% and 4.1% in the South and Southeast regions, respectively, while the North and Northeast regions reached 14.0% and 17.8%. Income disparities contribute to persistent inequalities in accessing quality services. Conversely, the North exhibits less demand due to its low population density. The judicial and executive administrative actions create regional profile variations based on decision structures and rationales, with a higher concentration of judicial demand in capitals due to greater availability of health and legal services (22, 35–37). Regional inequality mirrors the delay of the SUS in deploying technological innovations (18, 38).

In line with World Bank projections, Brazil remains highly unequal, with the Gini coefficient in 2021 revealing that the wealthiest 1% holds approximately half of the national wealth (37). According to World Health Organization estimates, in 2017, approximately one billion people allocated over 10% of their household budget to health expenses, with around 30% of them spending more than 25% of their income (7). The 2019 National Household Sample Survey found that the Central-West had the lowest overall access rate, while the South region had the highest. This was determined by the proportion of people who received at least one prescribed medication from the public healthcare service during their last healthcare visit, categorized by age groups. For the 0–17 age group, the national average was 29.80%, a figure lower than the percentage of individuals in this study who lacked direct public service access but obtained their claims through judicial intervention (39). Boing et al., noted that from 2013 to 2019, two-thirds of individuals in Brazil had to self-finance their medications due to challenges in acquiring them from the SUS (35). This study takes an economic perspective, illustrating that the ability to obtain these items, regardless of their source, correlates with an individual’s purchasing power. The rise in out-of-pocket expenses negatively impacts the potential for equitable financing in the healthcare system (13, 40).

In terms of pediatric medical specialty demand in Brazil, neurology and neurosurgery were predominant, especially in the South. Regarding this fact, it was shown that diseases of the nervous system also prevailed in the South and Southeast regions, in the lawsuits granted for the provision of medications to adult litigants, whose drugs were on the public lists of acquisition by SUS (20, 41–44). Nutritionist, endocrinologist, psychiatrist were also in high demand in Brazil and provide outpatient care for chronic conditions. An evaluation of the long-term impact of health spending revealed that multiple chronic and complex conditions in children and adolescents imply high use of the care network and outpatient services are driving a longitudinal trend of rising health spending (45).

The main criterion for granting by the magistrate was the identification of the need for access to the medication or procedure, supported by the plaintiff’s medical report. However, it is often observed that prescribers are unaware of the pharmaceutical products offered by SUS, prescribe non-funded medications (46), and those with higher costs, or even off-label use, a factor identified in a previous study as an “effective therapeutic safety failure” (36). The TSO allows evaluating the demands, determining allowable dispensations in public lists, and identifying where the claims can be obtained, among other details (47). An analysis by the National Council of Justice and the Institute of Education and Research conveys the same perception regarding the decision-making process of judges in specific cases supported by expert opinions (48), that the quality, measured by the percentage of court rulings based on scientific evidence, was compromised due to the limited use of expert opinions.

The individual dimension is predominant, addressing the unique needs of the claimants, leading to disparate treatment for those benefiting from the SUS. Public civil actions should extend access guarantees for the community, but they occur in smaller numbers than those that give individual guarantees to litigants, similar data occurred in the study by Paim et al. (43), which suggests that the type of legal representation is influenced by education and personal income, which would determine the individual character of lawsuits.

Verification of inadequate personal funds for treatment relies notably on self-declaration (18, 22, 36). Self-declared economic vulnerability provides a factual basis for gratuitous approval of claims by the judiciary in the name of social rights. Universality, achieved based on the frequency of accepted court-processed claims, lacks full assurance of the equality principle, as it does not equally extend to those in similar situations who did not seek legal action (18, 35).

Arguments associated with financial risk, such as the “existential minimum,” the “reserve of the possible” and the “solidarity among federated entities” were used in the text of the decisions on the right to health, in a similar way in the two periods defined in the analysis. The principle of “solidarity among public entities” means the responsibility of planning public spending on health provision divided among the three spheres of government. Even before the application of Law 13.655/2018, the Federal Supreme Court already signaled a rigorous examination of the claims and the evidence presented to the judge, to ensure that the peculiarities of the case to be judged are contemplated without negative impacts for the budgetary condition of the State (49).

The “minimum existential” are essential conditions for human survival supported by the constitutional right to health, reinforced the partial acceptance of the demands and corroborated with the favoring of the individual plaintiff, but the limited budgetary conditions of the State for collective demands were disregarded, even after the validity of Law 13.655/2018, which guides to correct the omission to the citizen equitably, according to their needs and singularities, without determining damages to the general interests (23, 24, 50).

The “reserve of the possible,” translate the financial availability to contemplate the claims request to the State, has often supported disputes involving various technologies with fluctuating costs, including medications and procedures offered by the SUS. However, studies often link the term to rare instances, high-cost claims, items not approved by Anvisa, or those not accounted for in budget forecasts (19, 20). Equity was not guaranteed by Law Number 13.655/2018, which is not mentioned in the decisions even after its enactment. The challenges and obstacles stemming from public policy demands are ignored, with the goal of reducing the risk of financial collapse for the healthcare system and its users.

Urgent implementation of policies is needed to guarantee equal access to health technologies and foster operational cooperation between public administration and the judiciary, reshaping the healthcare litigation process in Brazil. These policies aim to prevent negligence of public authorities in adhering to court rulings, ensuring that children are not denied healthcare access and receive “the highest possible standard of health” (5, 14, 36). Support is advocated for improved access, especially for individuals who frequently use services and lack health coverage (45, 51). In their study, Halfon et al. (51) called for actions aimed at families and communities, bolstered by structural determinants, to lessen health-related disparities among children. Reducing disparities requires the provision of comprehensive and quality assistance, so that it can have a positive impact in the long term on child and youth health, safeguarding the fair and ethical costs of the health system (15, 52).

There were notable limitations in jurisprudence repositories due to the lack of standardization in public access to court portals, impeding data collection. The presence of actions under seal introduced bias due to their unavailability, limiting the sample size to the convenience of available case laws. This was due to confidentiality issues determined as “excessively broad or unjustified” (53). Cohen and Bagley corroborated this observation, noting restricted access to health litigation information (54).

The scarcity of publications on health litigation involving children made it challenging to conduct a comprehensive analysis of the phenomenon and necessitated a comparison with studies involving adults. However, this study has strengths, such as selecting the target age group, having a nationwide scope, and employing a methodological approach supported by the normative paradigm, which allowed for comparing regional variability across the analyzed periods and may guide future research and public policies regarding health litigation.

## Conclusion

The study highlighted that the Brazilian courts did not adapt to the new decision-making model after the normative change instituted in Brazil in 2018. The judicial decisions continued to focus on individual demands, based on traditionally used legal guarantees and without dialog with the federated entities. Therefore, it emphasizes the importance of improving the decision-making process related to the access of litigants, both in the judicial and managerial scope.

The decisions must be supported by updated legislation, prioritizing technical-scientific evidence and respecting the predominant profile of health-related demands. This intersection highlights the interdisciplinary nature of the approach to health challenges and the importance of integrating legal, policy and research perspectives in the formation of effective health systems. Changes in legislation, such as the implementation of new normative models, can significantly affect the overall picture of health care provision. Legal scholars are encouraged to conduct research on the integration of law into the daily operations of legal professionals, with a special focus on the time required for the assimilation of changes.

The analyzed jurisprudence consist of relevant alternative sources to subsidize information about the guarantee of coverage of essential services for health care, because the official consolidated data, derived from the services performed at the health care points, do not reflect information about those people who did not use them due to lack of access to the care offered (55, 56).

Additionally, providing comprehensive, equitable, and high-quality care to children and adolescentes, including access to professionals who can guarantee essential health information is a “fundamental tool” for “meeting their physical needs” and promoting the growth of healthy adults, which positively impacts the country’s social and economic development, since it is a remarkable fact that this population group is disadvantaged from the moment of birth, due to differences in the concentration of resources and the low investment of public policies (6, 52, 57–59).

## Data availability statement

The original contributions presented in the study are publicly available. This data can be found here: https://doi.org/10.6084/m9.figshare.26065849.

## Ethics statement

Ethics approval statement is not required, patients and/or the public were not involved in the design, or conduct, or reporting, or dissemination plans of this research. Patient consent for publication is not applicable. This study did not require approval from the Research Ethics Committee (CEP) or the National Research Ethics Committee (CONEP), in accordance with the National Health Council Resolution 510/2016. The research used a freely accessible database, containing public domain information managed by the Brazilian Courts of Justice, with no restrictions for researchers or citizens. This study complies with the Access to Information Law Number 12.527, dated 18 November 2011, which promotes transparency of public information held by the Direct and Indirect Public Administration, including sentences published by the Judiciary. Furthermore, article 5, LX, of the 1988 Constitution stipulates that “procedural acts of a legal action are public, except when publicity restriction is required for privacy defense or social interest.” Thus, only information classified as confidential by the court restricts free access to researchers on the State Courts of Justice websites.

## Author contributions

SC: Conceptualization, Data curation, Formal analysis, Investigation, Methodology, Software, Writing – original draft, Writing – review & editing. PT: Conceptualization, Data curation, Investigation, Methodology, Software, Validation, Visualization, Writing – original draft, Writing – review & editing. RA: Conceptualization, Supervision, Validation, Writing – review & editing. VB: Conceptualization, Supervision, Validation, Writing – review & editing.
